# Targeted community screening in areas of high socioeconomic deprivation enhances the detection of liver fibrosis

**DOI:** 10.1097/HC9.0000000000001036

**Published:** 2026-09-16

**Authors:** Huw F. Purssell, Stephanie R. Landi, Alexander A. Warner-Levy, Lucy R. Bennett, Jennifer A. Scott, Christopher E. Mysko, Deepankar Gahloth, Oliver E. Street, Indra Neil Guha, Neil A. Hanley, Karen Piper Hanley, Varinder S. Athwal

**Affiliations:** 1 Division of Diabetes, Endocrinology and Gastroenterology, Faculty of Biology, Medicine & Health, University of Manchester, Manchester Academic Health Science Centre, Manchester, UK; 2 Manchester University NHS Foundation Trust, Manchester, UK; 3 NIHR Nottingham Biomedical Research Centre (BRC), Nottingham University Hospitals NHS Trust and University of Nottingham, Nottingham, UK; 4 College of Medical & Dental Sciences, University of Birmingham, Birmingham, UK; 5 University Hospitals Birmingham NHS Foundation Trust, Birmingham, UK; 6 Manchester Cell-Matrix Centre, Faculty of Biology, Medicine & Health, Manchester Academic Health Science Centre, University of Manchester, Manchester, UK

**Keywords:** diagnosis, hepatic steatosis, liver fibrosis, social determinants of health

## Abstract

**Background:**

Risk-factor-based community screening has been used to identify early liver disease. Risk-factor prevalence and liver-related outcomes are significantly affected by socioeconomic deprivation. However, the impact on targeted community screening is unknown. We evaluated the influence of deprivation on fibrosis detection within a population screening pathway.

**Methods:**

A total of 1613 patients were assessed using 2 pathways: Proactive case finding at 7 primary care practices (PCPs) and a comparator standard-of-care pathway. Individuals with specific risk factors were identified: obesity (BMI >30 kg/m^2^), type 2 diabetes, alcohol consumption >400/>280 g/week in males/females, respectively, coding for steatosis and ALT >50/>35 IU/mL in males/females, respectively. Liver stiffness measurement (LSM) ≥8.0 kPa was used to determine fibrosis, and the Index of Multiple Deprivation (IMD) was used to determine deprivation at the individual and neighborhood level.

**Results:**

In all, 325 (20.1%) patients had LSM ≥8.0 kPa. Fibrosis detection increased with the number of risk factors [2: OR 2.47 (*p*=0.027, 95% CI 1.11–5.50), 3: OR 5.19 (*p* <0.001, 95% CI 2.34–11.53), 4: OR 10.19 (*p* <0.001, 95% CI 4.44–23.40), 5: OR 23.40 (*p* <0.001, 95% CI 6.15–89.04)]. Patients with a low IMD score (<10) had significantly lower median LSMs than those with a high IMD score (>50) [5.4 kPa vs. 6.0 kPa (*p*=0.0005)]. In the proactive case finding pathway, obesity prevalence was higher in PCPs in areas of high deprivation [83.5% vs. 65.6% (*p*=0.003)]. Fibrosis detection was significantly higher in PCPs situated in neighborhoods with high socioeconomic deprivation compared with low deprivation [27.0% vs. 8.6% (*p*=0.028)].

**Conclusion:**

Liver fibrosis detection was disproportionately higher in neighborhoods with greater socioeconomic deprivation. Screening strategies targeting areas with higher deprivation could increase the diagnostic yield of advanced fibrosis.

## Graphical abstract

**Figure:**
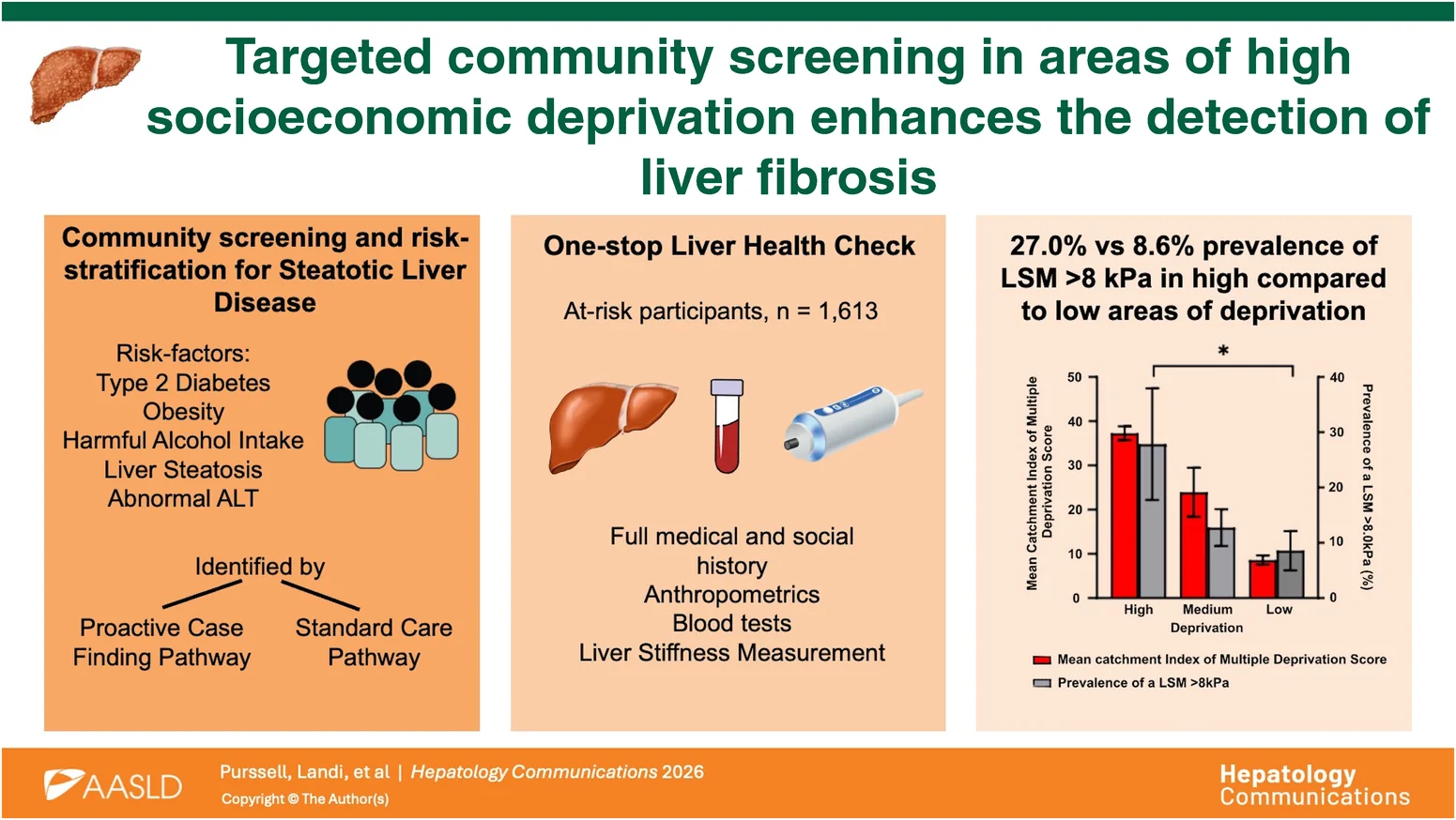

## INTRODUCTION

Liver disease is increasingly recognized as a silent epidemic; progressive, preventable, and shaped by deep structural inequalities.^1^ Socioeconomic deprivation is one of the strongest predictors of poor liver outcomes, including cirrhosis, HCC, and premature liver-related mortality globally.^2^^,^^3^ These disparities reflect more than differences in individual behaviors; they expose persistent gaps in access to early diagnosis and preventive care.

The preceding 10 years have seen further widening of health inequalities, particularly in underserved communities where multimorbidity, poor living conditions, and limited access to healthcare intersect.^4^ Liver disease follows this pattern.^2^ Although non-invasive diagnostics and risk-based screening pathways exist, their implementation has been uneven, often failing to reach the populations most at-risk.^5^^–^^7^ As a result, liver disease is frequently diagnosed late, when options for intervention are limited, and outcomes are poor.^8^

Steatotic liver disease (SLD), the most prevalent form of chronic liver disease (CLD), exemplifies these challenges.^9^ Its incidence continues to rise, driven by increasing rates of obesity, alcohol consumption, and type 2 diabetes mellitus (T2DM), all of which are more common in socioeconomically deprived populations.^1^ Without integrated early detection strategies that reach these communities, disparities in liver disease outcomes are likely to grow. Recently, a multi-national population screening study demonstrated 1.6% yield of advanced liver disease.^10^ However, given the health cost of such approaches, targeting higher-risk populations may be more financially prudent. Whether prioritizing areas of greater socioeconomic deprivation would increase fibrosis detection remains untested.

Greater Manchester, a region in the North West of England with high levels of deprivation and liver-related morbidity, has trialed an early detection pathway through the Integrated Diagnostics for Early Detection of Liver Disease (ID-LIVER) project. This collaboration between academic institutions, the National Health Service, and industry partners developed a community-based liver health assessment pathway, delivering non-invasive diagnostics directly into primary care and underserved areas.

While designed within the United Kingdom (UK) healthcare context, this approach provides an opportunity to address a knowledge gap for regions globally that face similar challenges in liver disease detection and health inequality. The primary aim of the study was to assess whether a targeted screening approach, comparing areas by levels of socioeconomic deprivation, would improve fibrosis diagnostic yield. This approach could inform scalable, equity-focused strategies for earlier diagnosis and improved outcomes in disadvantaged populations. Secondary aims were to understand the association between socioeconomic deprivation and fibrosis detection and compare targeted screening against the current standard-of-care pathways.

## METHODS

### ID-LIVER

In all, 1613 patients were prospectively recruited to the ID-LIVER study (https://sites.manchester.ac.uk/id-liver/) in Greater Manchester, UK, between November 2020 and August 2024. Ethical approval was granted by the Research Ethics Committee (REC) North of Scotland (Integrated Research Application System ID: 273633; REC reference: 20/NS/0055). The project was carried out in accordance with relevant guidelines and the Declaration of Helsinki and Istanbul. Written consent was provided by all participants. Inclusion criteria included patients with T2DM, obesity [body mass index (BMI) >30 kg/m^2^], harmful alcohol consumption (>280 g/week in females or >400 g/week in males), and abnormal serum ALT or liver steatosis on previous imaging. Patients under 18 years old, pregnant, breastfeeding, or with known CLD were excluded.

There were 2 recruitment pathways: the Standard-of-Care Pathway and the Proactive Case Finding Pathway. The Standard-of-Care Pathway (hereafter, the Reactive cohort) was similar to traditional referral processes into secondary care. General practitioners identified individuals with steatotic liver disease of any etiology and referred them for secondary care assessment, without requiring a non-invasive risk stratification, such as fibrosis-4 (FIB-4). Patients referred to 2 secondary care hospital sites throughout the duration of the study were offered recruitment. Patients with exclusion criteria or a putative diagnosis of an alternative liver disease were triaged straight to the secondary care hepatology clinic.

The Proactive Case Finding Pathway (hereafter, Proactive cohort) used coded risk factors to identify patients from their primary care records. Systemized Nomenclature of Medicine (SNOMED) codes were used for the following risk factors: T2DM, obesity (BMI >30 kg/m^2^) measured in the preceding 24 months, harmful alcohol consumption measured in the preceding 24 months, serum ALT >80 IU/L measured in the preceding 24 months, and a coding of liver steatosis. Primary care practices (PCP) searched electronic patient records using these codes to identify patients. Patients who met the inclusion criteria were invited to a community liver assessment clinic close to their home address. Patients were contacted twice by their PCP by postal, telephone, or electronic correspondence based on PCP preference and the project’s patient and public involvement consultation. Those who opted in were offered an appointment, which was predominantly carried out in a mobile clinical research unit. Patients who did not respond to 2 invitations were defined as non-responders.

At the clinic, the pathways merged, and a clinical history and blood tests for a liver etiological screen were taken. Patients were asked to fast for 3 hours before their appointment, and Vibration-Controlled Transient Elastography (VTCE) (Fibroscan, Echosens, Paris) was performed to obtain a liver stiffness measurement (LSM) in kilopascals (kPa). The M and XL probes were available and used alongside automated probe selection technology throughout the study. A valid LSM was defined as an IQR/Median <30%. An 8.0 kPa LSM cut-off was used to discriminate between patients with and without fibrosis, as per guidance for community detection.^11^

Occupation status data was collected as either employed, unemployed, or retired. Occupations were grouped by the Standard Occupation Classification 2020 (SOC2020). SOC2020 is used in the United Kingdom to classify jobs by skill level and content, and categorizes occupations into 9 levels.^12^

The ID-LIVER dataset was curated on Microsoft Access 2016 (Microsoft, USA) with statistical analysis performed using GraphPad Prism version 10 (GraphPad, USA), SPSS version 29 (IBM, USA), and R version 4.4.3 (R Foundation for Statistical Computing, Austria).

### Index of multiple deprivation

The Index of Multiple Deprivation (IMD) is an official UK measure of relative deprivation. It consists of 7 domains: income, employment, education and skills, health and disability, crime, barriers to housing and services, and the living environment. Each subdomain is weighted, with income and employment being the most important, followed by education and skills, then health and disability. IMD is calculated by the UK Office for National Statistics at a Lower layer Super Output Area (LSOA) level: a defined area with a similar population size, typically 1500 residents or 650 households. LSOA data was obtained using the patient’s home postcode through publicly available data sources (https://imd-by-postcode.opendatacommunities.org/imd/2019). Higher IMD scores represent increased deprivation (eg, the IMD scores for the most and least deprived LSOA are 92.735 and 0.541, respectively).

UK neighborhood-level data can also be assessed at a ward level. Wards are electoral districts; there are over 200 within Greater Manchester with an average population of 17,248. IMD scores at a ward level are an aggregate of the LSOAs within that ward.^2^^,^^13^ Neighborhood-level analysis of fibrosis detection and deprivation for the whole cohort was performed at a ward level. Wards with ≥10 participants were split into equal quartiles based on fibrosis detection rates.

To understand the distribution of liver fibrosis across the socioeconomic spectrum, the Wagstaff concentration index $\left(CI_{w}\right)\;$ was used. The concentration index is a useful measurement of health inequality.^14^^,^^15^ Participants were ranked from the most to least deprived using IMD, and the cumulative proportion with a LSM ≥8.0kPa was incrementally plotted. The $CI_{w}\;$ is a value between 1 and −1. If there is equality, the index will be 0, and the concentration curve is linear (ie, the more participants tested, the more disease is detected, regardless of socioeconomic deprivation). If ill-health is concentrated among individuals living in deprived neighborhoods, the index will be closer to −1, and the concentration curve will have a left shift. This method provides a numerical value demonstrating that the proportion of a variable of interest (in this case, the presence of liver fibrosis) is skewed in relation to socioeconomic deprivation. This was performed for the whole cohort and for individuals with harmful alcohol intake, T2DM, and obesity. To understand the contribution to health inequality of these variables and the residual unexplained health inequality, a post hoc decomposition was undertaken.

### Risk factors geo-visualization in Greater Manchester

The Greater Manchester Dataset (GM-D) contains variables held within the Greater Manchester Care Record for each individual. This contains demographics, past medical history, medications, and results from within primary care records across Greater Manchester.^16^ SNOMED codes for medical history and recorded clinical variables over the last 24 months were used to identify records with risk factors for SLD. (Supplemental Table S1, https://links.lww.com/HC9/C470). The GM-D was also used to visualize deprivation for each ward and to establish a denominator for the prevalence of ID-LIVER recruitment by ward. The data analytics team at Health Innovation Manchester collated the GM-D within a secure data environment. To preserve confidentiality, individual-level data is aggregated by ward. Categorical variables are summarized as prevalence, while non-categorical variables are represented as a mean.

### PCPs in the proactive pathway

**FIGURE 1:**
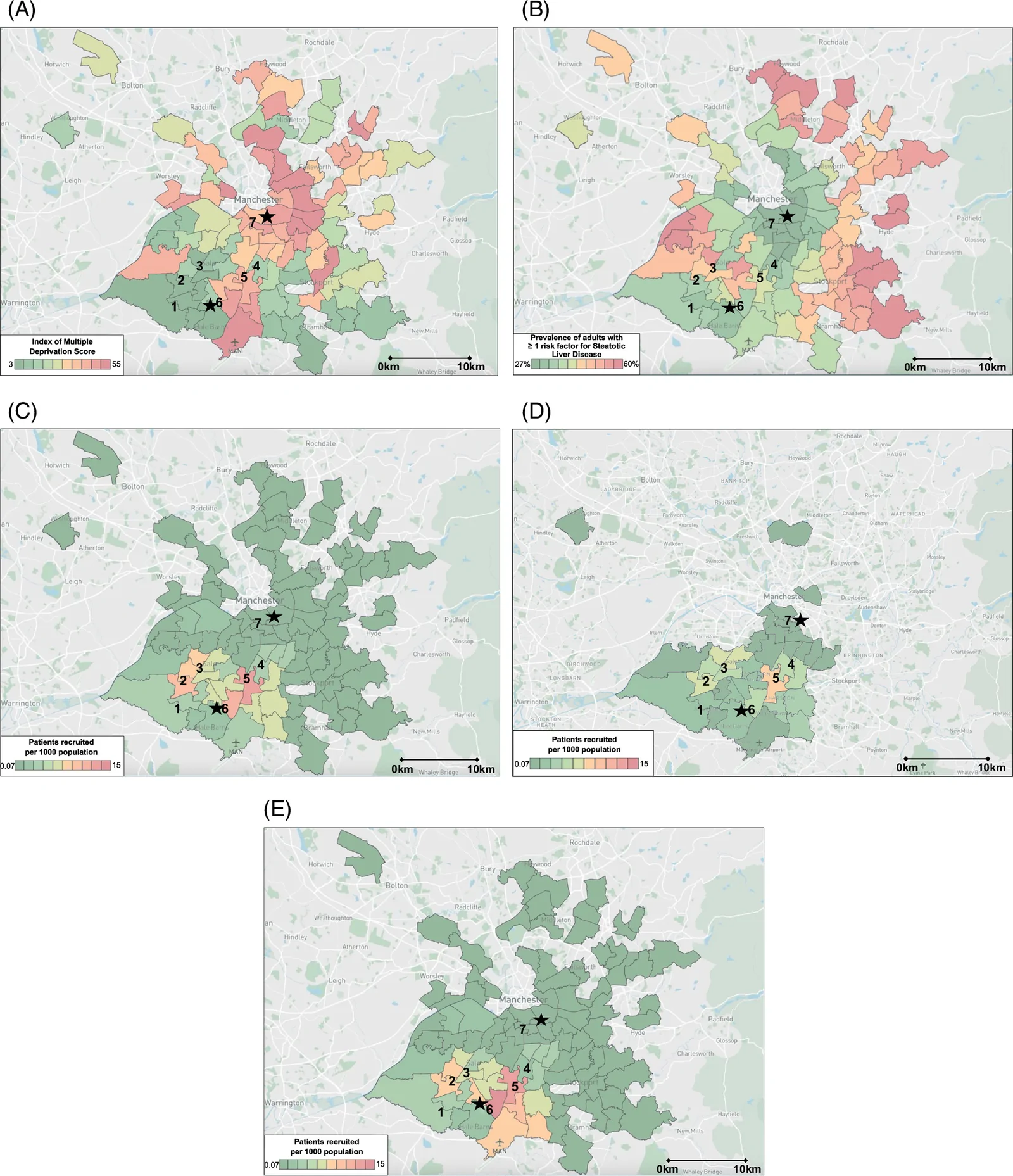
Cartographic heat maps of ward-level deprivation, risk-factor prevalence, and recruitment to ID-LIVER per 1000 adult population, in Greater Manchester. (A) The Index of Multiple Deprivation (IMD); (B) prevalence of adults living within each ward with ≥1 liver-related risk-factor; (C) total ID-LIVER cohort; (D) proactive cohort; (E) reactive cohort. Stars show the local secondary care hospital sites. Markers 1–7 represent the locations of primary care practices (PCPs) undergoing proactive case finding. Abbreviations: ID-LIVER, Integrated Diagnostics for Early Detection of Liver Disease; IMD, Index of Multiple Deprivation; PCPs, primary care practices.

PCPs in neighborhoods of varying deprivation were identified utilizing anonymized primary care data from the Greater Manchester Care Record and recruited by direct contact (Table 1 and Figure 1A). As PCPs often span multiple wards, PCP catchment IMD scores were calculated by curating the mean IMD score from all LSOAs within the practice catchment area. This allowed for a more precise categorization of PCPs in high, medium, and low-deprivation neighborhoods (Table 1).

**TABLE 1:** List of primary care practices (PCPs) involved in community proactive case finding in order of PCP catchment IMD score

Key (PCP)	Catchment IMD score	Total population	Proactive patients recruited (n)	Mean proactive cohort IMD score (SD)	≥1 Risk factor (% PCP population)	1 Risk factor (% PCP population)	2 Risk factors (% PCP population)	≥3 Risk factors (% PCP population)	Practice deprivation classification
1. (St. John's Medical Centre)	7.61	12,689	71	8.65 (8.50)	1541 (12.1)	1208 (9.5)	279 (2.2)	54 (0.4)	Low
2. (West Timperley Medical Centre)	8.59	5956	65	11.03 (8.7)	865 (14.5)	679 (11.4)	165 (2.8)	21 (0.4)	Low
3. (Washway Road Medical Centre)	9.66	11,704	119	12.90 (9.5)	1898 (16.2)	1442 (12.3)	383 (3.3)	73 (0.6)	Low
4. (Didsbury Medical Centre)	20.02	11,376	89	19.36 (10.99)	1000 (8.8)	807 (8.8)	170 (1.5)	23 (0.2)	Medium
5. (Northenden Group Practice)	27.85	8393	207	29.75 (15.44)	2181 (26.0)	1635 (18.7)	414 (4.7)	132 (1.5)	Medium
6. (The Maples Medical Centre)	38.39	6542	91	41.36 (16.68)	1356 (20.7)	1036 (15.8)	295 (4.5)	25 (0.4)	High
7. (The Arch Medical Centre)	36.17	10,242	34	42.13 (15.33)	1153 (11.3)	898 (8.8)	220 (2.1)	35 (0.3)	High

*Note:* The practice key corresponds to the location markers in Figure 1A.

Abbreviation: IMD, Index of Multiple Deprivation; PCP, primary care practices.

## RESULTS

A total of 1613 patients were recruited between September 2020 and August 2024: 697 (43.2%) and 916 (56.8%) to the proactive and reactive cohorts, respectively. Of these, 829 (51.4%) were male with a mean age of 56 (SD ±13.83) years. A total of 1306 (81%) patients were of White ethnicity, and the mean BMI was 32.3 (SD ± 6.9) kg/m^2^. Of these, 516 (32.0%) had T2DM and 257 (15.9%) drank harmful quantities of alcohol. A total of 1065 (66.0%), 182 (11.3%), and 208 (12.9%) patients were diagnosed with metabolic dysfunction–associated steatotic liver disease (MASLD), metabolic dysfunction- and alcohol-associated liver disease (MetALD), and alcohol-associated liver disease (ALD), respectively. A total of 325 (20.1%) had an LSM >8.0 kPa (Table 2).

**TABLE 2:** Total proactive and reactive cohorts characteristics and demographics

Characteristic	Whole cohort (n=1613)	Proactive pathway (n=697)	Reactive pathway (n=916)
Age (years), mean (SD)	56 (13.8)	57.5 (13.2)	54.8 (14.2)
Male, n (%)	829 (51.4)	361 (51.8)	468 (51.1)
Ethnicity, n (%)			
White	1306 (81.0)	592 (84.9)	714 (78.0)
South Asian	182 (11.3)	54 (7.7)	131 (14.3)
Black	65 (4.0)	27 (3.9)	43 (4.7)
Other (mixed, Chinese, or unknown)	60 (3.7)	24 (3.4)	27 (3.0)
Index of Multiple Deprivation, mean (SD)	27.3 (18.9)	23.6 (17.3)	30.8 (19.6)
Liver-associated risk factors, n (%)			
Harmful alcohol consumption	257 (15.9)	117 (16.8)	140 (15.3)
T2DM	516 (32.0)	268 (38.5)	248 (27.1)
Abnormal ALT	674 (41.8)	217 (31.1)	457 (49.9)
Obesity	978 (60.6)	463 (66.4)	515 (56.2)
Steatosis	1079 (66.9)	208 (44.2)	871 (95.1)
BMI (kg/m^2^), mean (SD)	32.3 (6.9)	32.6 (6.1)	32.1 (7.5)
HbA1c (mmol/mol), mean (SD)	42.9 (15.6)	43.8 (14.0)	42.9 (16.1)
ALT (IU/L), mean (SD)	48.1 (36.5)	41.7 (34.5)	53.3 (37.1)
AST (IU/L), mean (SD)	41.6 (35.9)	35.6 (31.2)	46.5 (38.5)
Platelets (×10^9^/L), mean (SD)	246.9 (76.2)	253.9 (74.9)	243.6 (73.1)
FIB-4, mean (SD)	1.56 (1.4)	1.42 (0.7)	1.68 (1.5)
VTCE probe, n (%)			
M	981 (60.8)	429 (61.5)	552 (60.2)
XL	606 (37.6)	257 (36.9)	349 (38.1)
Unreliable scan	26 (1.6)	10 (1.4)	15 (1.6)
LSM measurements, n (%)			
>8.0 kPa	325 (20.1)	91 (13.0)	233 (25.4)
>15.0 kPa	95 (5.9)	21 (3.0)	77 (8.4)
Number of liver-related risk factors, n (%)			
0	88 (5.5)	0	88 (9.6)
1	369 (22.9)	217 (31.1)	152 (16.5)
2	557 (34.5)	370 (52.9)	187 (20.4)
3	419 (26.0)	102 (14.6)	317 (34.6)
4	162 (10.0)	8 (1.1)	154 (16.8)
5	18 (1.1)	0	18 (2.1)
Liver diagnosis, n (%)			
MASLD	1065 (66.0)	464 (66.6)	601 (65.6)
MetALD	182 (11.3)	79 (11.3)	102 (11.1)
ARLD	208 (12.9)	59 (8.5)	149 (16.2)
Other	93 (5.8)	40 (5.7)	51 (5.5)
None	66 (4.1)	55 (7.9)	10 (1.1)

*Note:* Parametric data are displayed as mean (±SD) and categorical data as n (%). Harmful alcohol intake was defined as >280 g/week (females) or >400 g/week (males). An abnormal ALT level >35 IU/L (females) or >50 IU/L (males). An unreliable VCTE was defined as an IQR/Median ≥30%.

Abbreviations: ARLD, alcohol-related liver disease; ALT, alanine aminotransferase; AST, aspartate aminotransferase; BMI, body mass index; FIB-4, fibrosis-4; HbA1c, hemoglobin A1c; IQR, interquartile range; LSM, liver stiffness measurement; MASLD, metabolic dysfunction–associated steatotic liver disease; MetALD, metabolic dysfunction- and alcohol-associated liver disease; SD, standard deviation; T2DM, type 2 diabetes mellitus; VCTE, vibration-controlled transient elastography.

Patients were recruited across Greater Manchester. Clinic coverage, and therefore patient recruitment, reflects secondary care referral patterns. From the GM-D, an estimated adult population of 1,099,829 adults lives within wards covered by recruitment, and 522,037 (47.5%) have ≥1 liver-related risk factor (Figures 1A–E).

A total of 369 (22.9%) patients had one risk factor, and 557 (34.5%) had 2 risk factors (Table 2). A total of 419 (26.0%) and 162 (10.0%) patients had 3 and 4 risk factors, respectively. Only 18 (1.1%) patients had 5 risk factors. No risk factors were identified in 88 (5.5%), as patients had modified their risk factors between identification and assessment. These patients were still recruited and included in data analysis to reduce selection bias, as this would reflect a real-world targeted screening pathway. There were no significant differences in mean IMD scores between patients with 1, 2, 3, and 4 or more risk factors (*p*=0.051). The likelihood of fibrosis detection increased with the number of risk factors. Patients with 2 [OR 2.47 (*p*=0.027, 95% CI 1.11–5.50)], 3 [OR 5.19 (*p* <0.001, 95% CI 2.34–11.53)], 4 [OR 10.19 (*p* <0.001, 95% CI 4.44–23.40)], and 5 [OR 23.40 (*p* <0.001, 95% CI 6.15–89.04)] risk factors were at significantly higher risk of fibrosis. Having one risk factor was associated with an increased, but not significant, risk of fibrosis detection (OR 1.29, *p*=0.552). T2DM [OR 1.92 (*p* <0.001, 95% CI 1.50–2.48)] and harmful alcohol consumption [OR 3.20 (*p* <0.001, 95% CI 2.40–4.27)] were associated with a significantly higher risk of fibrosis.

### Deprivation correlates with fibrosis detection

**FIGURE 2:**
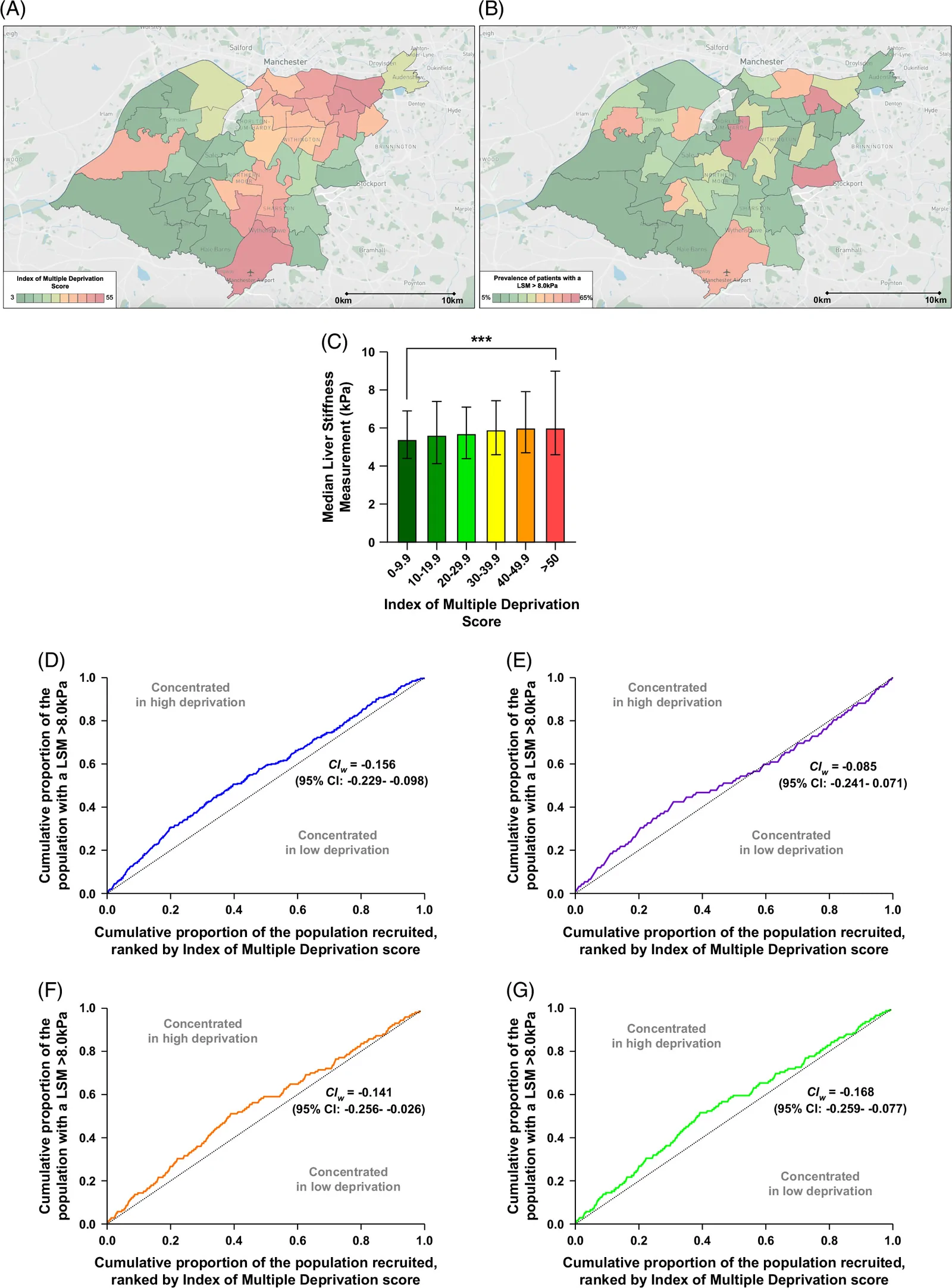
Presence of fibrosis by the Index of Multiple Deprivation (IMD) score. (A, B) Heat maps of Greater Manchester at a ward level, for wards where ≥5 patients were recruited from ID-LIVER. A: Ward IMD score, red represents a higher Index of Multiple Deprivation (IMD) score and deprivation. B: Prevalence of a liver stiffness measurement (LSM) >8.0 kPa in patients recruited to the ID-LIVER cohort. Red represents a higher fibrosis prevalence. Higher fibrosis detection was observed in wards with increased deprivation (*r* ^2^=0.24, *p*=0.002). (C) Median LSM in individual patients, grouped by IMD score, in the ID-LIVER cohort. Individuals with high socioeconomic deprivation (IMD >50) had a significantly higher median LSM than the least socioeconomically deprived individuals (IMD <10) [6.0 kPa vs. 5.4 kPa (*p*=0.0005)]. There were no significant differences between the groups (*p*=0.078). Error bars represent the interquartile range; ***p<0.001. (D–G) ID-LIVER cohort health equality concentration curves: D: Whole ID-LIVER cohort [the Wagstaff concentration index (*CI_w_*)=−0.156 (95% CI −0.229 to −0.098)], E: Harmful alcohol consumption [*CI_w_* =−0.085 (95% CI −0.241 to 0.071)], F: Type 2 diabetes [*CI_w_* =−0.141 (95% CI −0.256 to −0.026)], G: Obesity (BMI >30 kg/m^2^) [*CI_w_* =−0.168 (95% CI −0.259 to −0.077)]. The central dotted line represents equality: when the curve is to the left of this line, an LSM >8.0 kPa is more concentrated in individuals living in neighborhoods with high deprivation. Abbreviations: BMI, body mass index; *CI_w_*, the Wagstaff concentration index; ID-LIVER, Integrated Diagnostics for Early Detection of Liver Disease; IMD, Index of Multiple Deprivation; LSM, liver stiffness measurement.

There were 37 wards with ≥10 patients recruited. Risk factors, fibrosis prevalence, and deprivation data are available in Supplemental Table S2, https://links.lww.com/HC9/C470. There were no differences in the prevalence of T2DM (*p*=0.695), obesity (*p*=0.661), or harmful alcohol consumption (*p*=0.534) between quartiles (Supplemental Table S2, https://links.lww.com/HC9/C470). Wards with a higher detection rate of fibrosis weakly correlated with a higher IMD (*r* ^2^=0.24, *p*=0.002). Heat maps show that wards with high deprivation (Figure 2A) had a higher detection rate of fibrosis (Figure 2B).

There was a significantly higher mean IMD score in patients with LSM 8.0–14.9 kPa (31.75) and >15.0 kPa (31.76) compared with patients with LSM <8.0 kPa (26.04) [*F*=(2, 1535)=11.77, *p*=<0.0001]. However, at an individual level, there was no significant correlation between IMD score and LSM (*r* ^2^=0.005) or FIB-4 (*r* ^2^=<0.001), and significance was only detected at extremes of socioeconomic deprivation status. Individual patients with a low deprivation (IMD score <10) had a significantly lower median LSM compared with the highest deprivation (IMD >50) [5.4 kPa vs. 6.0 kPa (*p*=0.0005)] (Figure 2C). There were no significant differences between the groups (*p*=0.078).

Despite this, the proportion of participants with liver fibrosis is concentrated in the socioeconomically deprived proportion of the cohort [*CI*_*w*_ −0.156 (95% CI −0.229 to −0.098)] (Figure 2D). This trend was mirrored in participants with T2DM and obesity (*CI*_*w*_ −0.141 (95% CI −0.256 to −0.026) and −0.168 (95% CI −0.259 to −0.077) respectively) (Figures 2F, G). Among individuals with harmful alcohol consumption, liver fibrosis was concentrated at the extreme of socioeconomic deprivation. However, this association reversed in the least deprived proportion of the cohort, suggesting a non-linear relationship between socioeconomic status and liver fibrosis [*CI*_*w*_ −0.085 (95% CI −0.241 to 0.071)] (Figure 2E). In the whole cohort, the residual concentration index was −0.129, after adjustment for T2DM, obesity, harmful alcohol consumption, age, and gender, and 95.0% of inequality in liver fibrosis prevalence remained unexplained by these factors alone (Supplemental Table S3, https://links.lww.com/HC9/C470).

### Subanalysis of the IMD domains

Subanalysis of the 7 domains of the IMD score used linear regression to compare domains to fibrosis detection rate at a ward level, and between quartiles. Multivariate analysis was also performed, adjusting for the prevalence of T2DM, obesity, and harmful alcohol consumption (Table 3).

**TABLE 3:** Correlation of the IMD subdomains and the prevalence of fibrosis in patients assessed

IMD subdomain	Unadjusted *r* ^2^	Adjusted *r* ^2^	*p*
Income	0.24	0.28	**0.032** ^a^
Employment	0.20	0.26	**0.042** ^a^
Education and skills	0.23	0.28	**0.029** ^a^
Health and disability	0.20	0.26	**0.026** ^a^
Crime	0.19	0.26	**0.042** ^b^
Barriers to housing and services	0.04	0.17	0.190
Living environment	0.10	0.13	0.334

*Note:* Multivariate analysis adjusted for the prevalence of T2DM, obesity, and harmful alcohol consumption.

^a^ *p*<0.05.

^b^ *p*<0.01.

Abbreviations: IMD, Index of Multiple Deprivation; T2DM, type 2 diabetes.

Across the income, employment, education and skills and health, and disability subdomains, the wards in the highest quartile for fibrosis detection had a significantly higher proportion of the population with a socioeconomic disadvantage: 0.22 versus 0.11 (*p*=0.035; 95% CI 0.01–0.18), 0.15 versus 0.09 (*p*=0.036, 95% CI 0.00–0.11), 10.75 versus 25.92 (*p*=0.022; 95% CI 2.51–27.83) and 0.90 versus 0.07 (*p*=0.380; 95% CI 0.05–1.61), respectively. This association remained significant in multivariate analysis after adjusting for T2DM, obesity, and harmful alcohol consumption: income: *r* ^2^=0.28 (*p*=0.032), employment: *r* ^2^=0.26 (*p*=0.042), education and skills: *r* ^2^=0.28 (*p*=0.029), and health and disability: *r* ^2^=0.26 (*p*=0.006) (Table 3).

To better understand whether the observed association between deprivation and fibrosis detection is driven by individual or societal socioeconomic deprivation, employment status, and occupation was recorded. There was a higher proportion of patients with LSM >8.0 kPa among those who were retired (23.6%) or unemployed (29.9%) compared with those employed (14.3%) (χ^2^=20.37, *p*<0.0001) (Supplemental Figure S1, https://links.lww.com/HC9/C470). Occupations were classified using SOC2020. Patients in professional occupations and associate professional occupations (SOC2020 groups 2 and 3) had the lowest incidence of LSM >8.0 kPa (8.5% and 9.5%, respectively). Although there was a significantly higher incidence of LSM >8.0 kPa in skilled trade occupations (group 5; 20.3%), leisure and service occupations (group 7; 24.4%) and process, plant and machinery operatives (group 8; 23.7%) (χ^2^=4.66, *p*=0.031) (Supplemental Figure S2, https://links.lww.com/HC9/C470), the likelihood of having an increased LSM was not significantly greater [group 5 OR 1.04 (*p*=0.930), group 7 OR 1.32 (*p*=0.571), and group 8 OR 1.27 (*p*=0.590)].

In the crime domain, although there was a higher risk of crime in wards in the highest quartile of fibrosis detection, this was not statistically significant [0.87 vs. 0.30 (*p*=0.071)]. However, multivariate analysis showed wards with higher levels of crime weakly correlated with increased detection of fibrosis (*r* ^2^=0.26, *p*=0.042).

The only 2 domains that showed no significant association with fibrosis detection were barriers to housing and services, and living environment. Barriers to housing and services measure the accessibility of housing and local services. Given the urban population of Greater Manchester, unsurprisingly, there was no correlation between this and fibrosis detection (*r* ^2^=0.17, *p*=0.190), and there were no differences between domain scores in wards in the highest and lowest quartiles (*p*=0.225). The living environment domain measures the quality of local living environments, both indoor (housing quality) and outdoor (air quality). There were no significant differences between wards in the highest quartile compared with the lowest quartile (*p*=0.364). There was no correlation between the living environment domain score and fibrosis detection in multivariate analysis (*r* ^2^=0.113, *p*=0.334). There was no significant association between fibrosis and the amount of green space in each ward (*r* ^2^=0.09). However, wards with a higher prevalence of fibrosis weakly correlated with worse air quality (*r* ^2^=0.16; *p*=0.015), and wards in the lowest quartile of fibrosis detection had better air quality; however, this was not statistically significant [0.75 vs. 0.53 (*p*=0.093)].

### The effect of deprivation on the proactive case finding pathway

#### Risk factors are present in all areas regardless of deprivation

**FIGURE 3:**
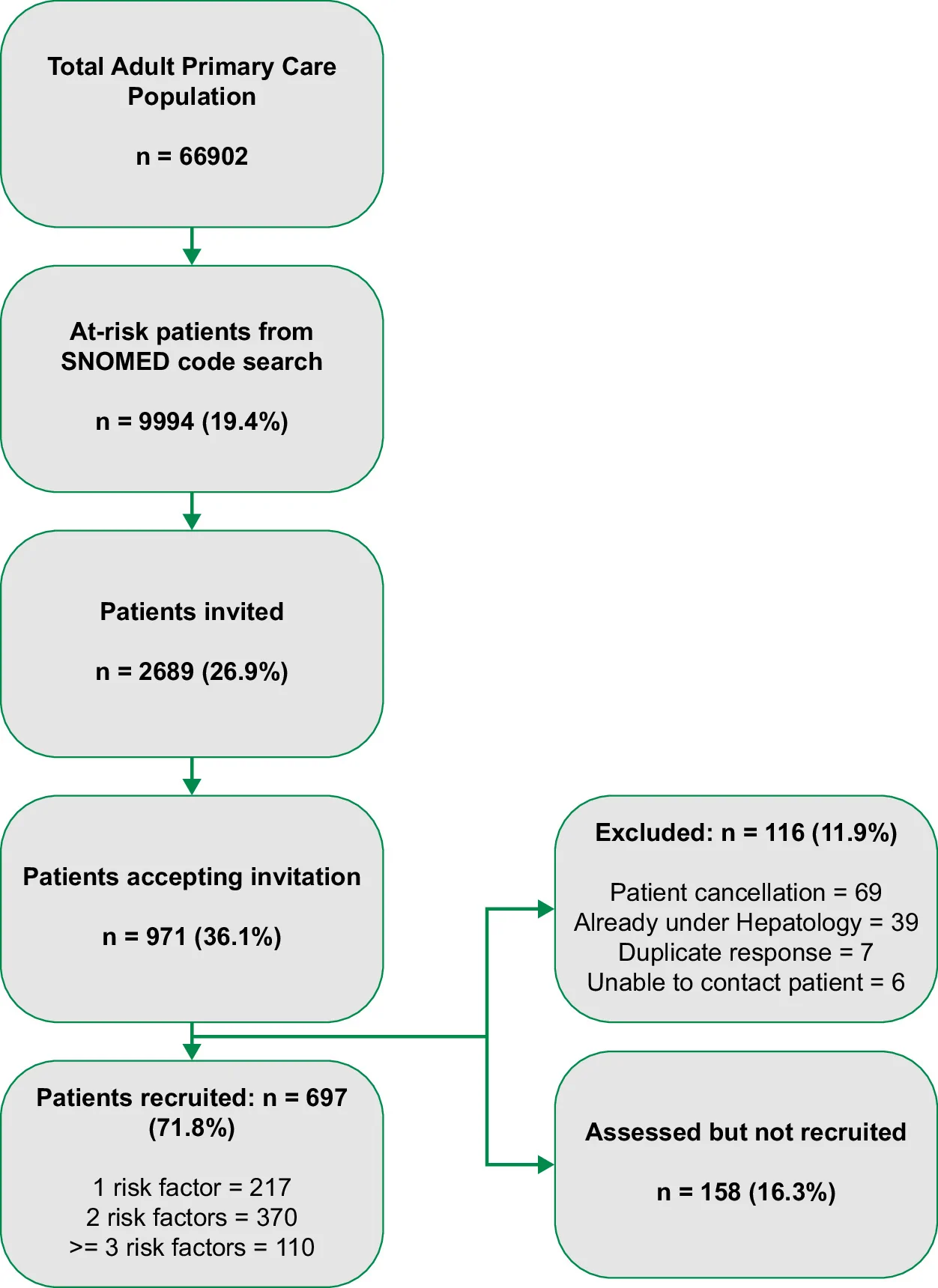
Flowchart showing the total adult population in the proactive pathway and the subsequent number of patients with risk factors—invited and recruited. Abbreviation: SNOMED, Systemized Nomenclature of Medicine.

Seven PCPs, with a total adult population of 67,243 patients, were involved with proactive recruitment, of which 9994 (14.9%) patients had at least one risk factor for liver disease (Figure 3). There were no significant differences in the proportions of patients with risk factors between practices located in areas of high, medium, and low deprivation [*F*(2, 3)=0.996, *p*=0.456].

Within study resource constraints, it was not feasible to assess every patient identified. Invitation was prioritized by risk factors identified: all patients with 2 or more risk factors were invited, and a random equal proportion with a single risk factor were invited. A total of 2689 patients with at least one risk factor were invited to make an appointment at a community liver assessment clinic. Of these invited, 971 (36.1%) of those invited opted in, 697 (23.3%) patients attended and were recruited (Figure 3). The uptake was in line with national screening invitations.^17^ The mean invite to recruitment by practice deprivation was high—4.8% (SD±2.66), medium—9.2% (SD±0.45), and low—6.1% (SD±1.46), without statistical difference (*p*=0.13) (Supplemental Table S4, https://links.lww.com/HC9/C470). For each PCP, there was no significant difference between catchment IMD and proactive cohort IMD, indicating representative PCP recruitment (Table 1). There was no significant difference between the mean number of risk factors identified between PCPs in neighborhoods of high (mean: 1.95), medium (mean: 1.87), and low deprivation (mean: 1.77) [*F*(2, 693)=2.79; *p*=0.062].

**FIGURE 4:**
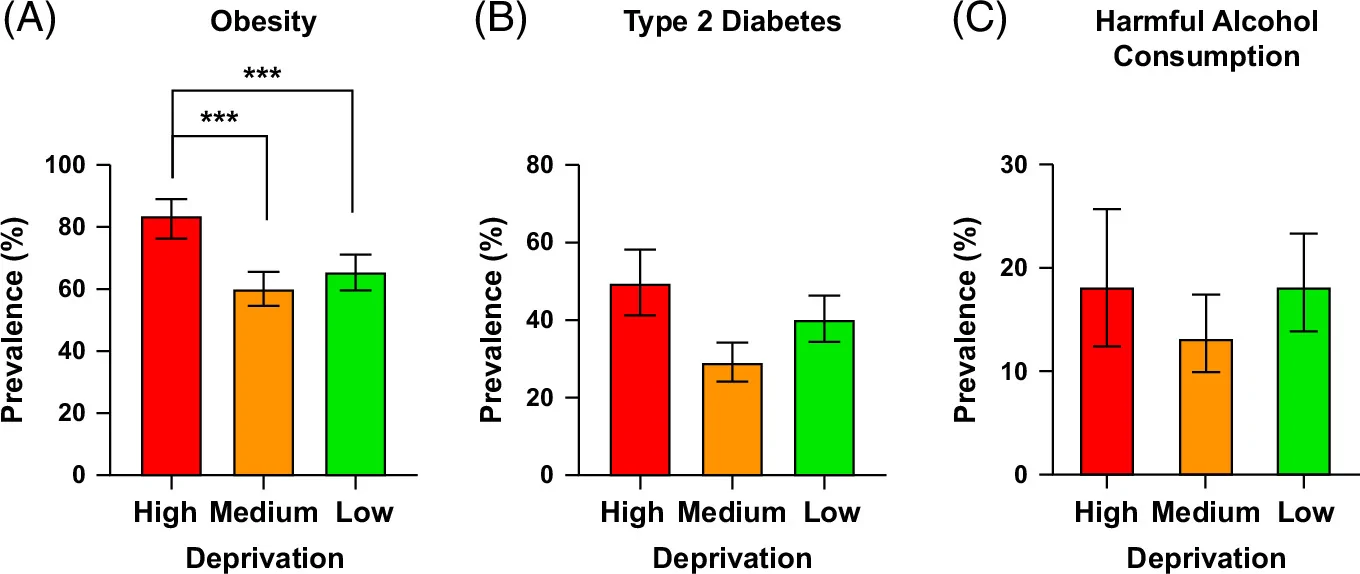
Bar charts showing the prevalence of risk factors in patients seen in primary care practices from areas of high, medium, and low deprivation. (A) Obesity (BMI >30 kg/m^2^), (B) type 2 diabetes (T2DM), and (C) harmful alcohol consumption. The prevalence of obesity was significantly higher in areas of high deprivation compared with areas of medium (*p*<0.0001) and low (*p*=0.0003) deprivation. There were no differences in the prevalence of T2DM (*p*=0.091) and harmful alcohol consumption (0.207) between the different areas. Error bars represent the 95% confidence intervals; ***p<0.001. Abbreviations: BMI, body mass index; T2DM, type 2 diabetes.

The prevalence of patients with obesity in areas of high deprivation (83.5%) was significantly greater than in areas of medium (60.3%) and low (65.6%) deprivation (*p*=<0.0001 and *p*=0.003-, respectively) (Figure 4A). Mean BMI was significantly higher in areas of high deprivation (34.7 kg/m^2^) compared with medium (32.0 kg/m^2^) and low (32.3 kg/m^2^) deprivation (*F*(2, 691)=9.760, *p* <0.001). The prevalence of patients with T2DM was higher in areas of high deprivation (50.4%) compared with medium (44.6%) and low (42.09%) deprivation; however, this was not statistically significant (*p*=0.091) (Figure 4B). There were no differences in the prevalence of harmful alcohol consumption as a risk factor (*p*=0.207) (Figure 4C). There were no significant differences in reported grams of alcohol consumption per week between the different areas (*p*=0.737).

#### Fibrosis case finding was more successful in socioeconomically deprived areas of higher deprivation

A total of 697 patients were recruited to the proactive cohort. Of these, 361 (51.8%) patients were male with a mean cohort age of 57.47 (SD ±13.2) years. A total of 592 (84.9%) patients were of White ethnicity, and 268 (38.5%) had T2DM. The mean BMI was 32.59 (SD ±6.1) kg/m^2^. A total of 117 (16.8%) patients consumed harmful quantities of alcohol. A total of 464 (66.6%), 79 (11.3%), and 59 (8.5%) patients were identified as having MASLD, MetALD, and ALD, respectively. There was no evidence of hepatic pathology in 55 (7.9%) patients. A total of 91 (13.1%) patients were identified as having a raised LSM (>8.0 kPa) [65 (71.4%) patients with MASLD or MetALD, 19 (20.9%) with ALD, 2 (2.2%) with primary biliary cholangitis, 3 (3.3%) with congestive hepatopathy, and 2 (2.2%) with an unclear etiology awaiting further investigations]. Ten (1.4%) patients had unreliable VCTE results (Table 2).

**FIGURE 5:**
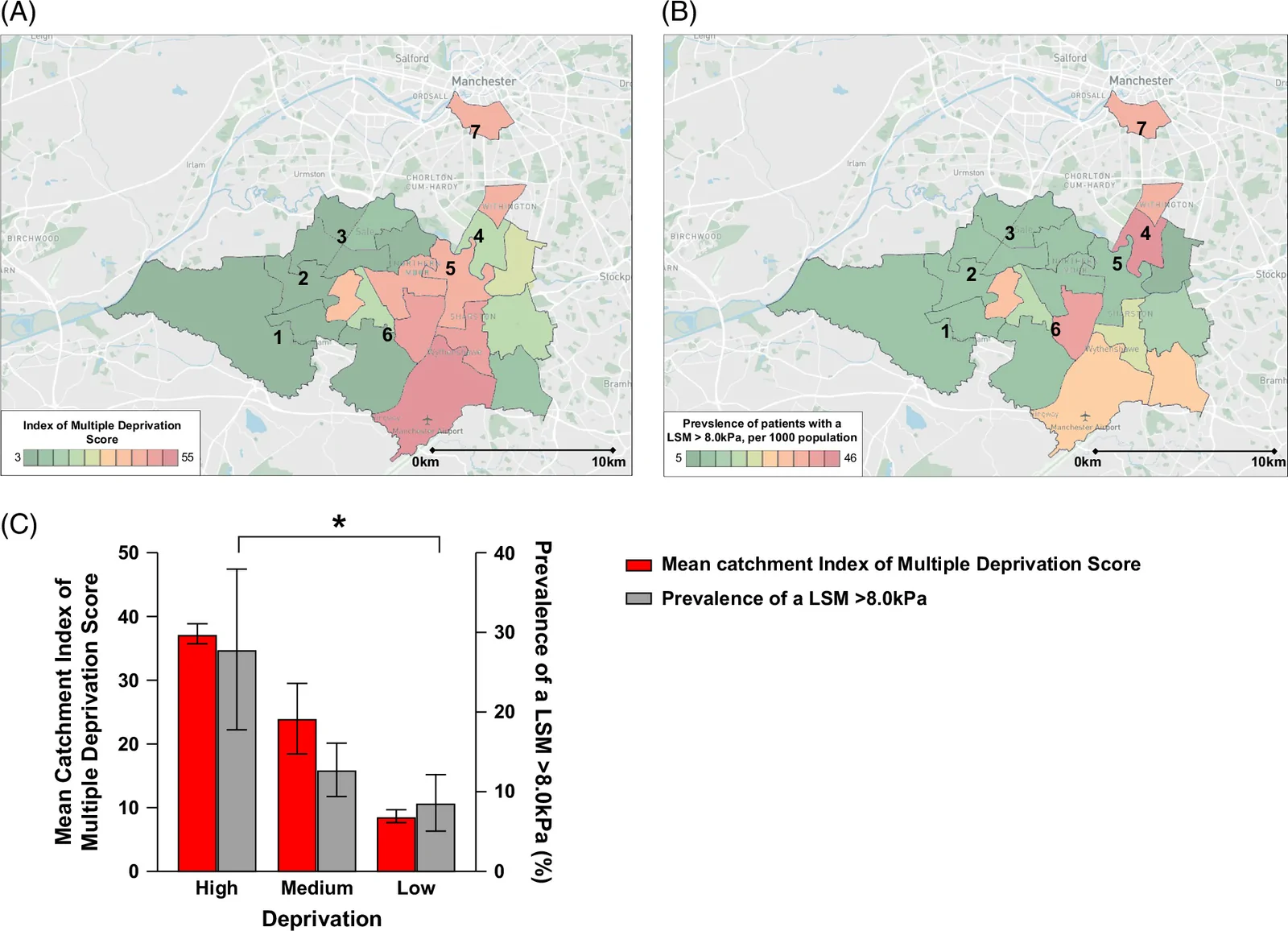
Impact of deprivation on the prevalence of fibrosis in patients recruited to the ID-LIVER proactive cohort. (A) Heat map showing the mean Index of Multiple Deprivation (IMD) by ward, red represents a higher IMD score. (B) Prevalence of liver fibrosis [liver stiffness measurement (LSM) >8.0 kPa] in the proactive cohort. Higher detection rates of fibrosis correlated with areas of higher deprivation (*r* ^2^=0.154; *p*=0.006). Markers 1–7 correspond to the primary care practice (PCP) key (Table 1). (C) Bar chart showing the mean catchment area for PCPs (red) and the mean number of patients identified with LSM >8.0 kPa. Error bars represent 95% confidence intervals. Fibrosis detection was significantly higher in areas of high deprivation compared with areas of low deprivation [27.0% vs. 8.6% (*p*=0.028, 95% CI 3.17–33.00)]; *p<0.05. Abbreviations: ID-LIVER, Integrated Diagnostics for Early Detection of Liver Disease; IMD, Index of Multiple Deprivation; LSM, liver stiffness measurement; PCP, primary care practice.

In the proactive cohort, there was no significant correlation between LSM and IMD score at an individual level (*r* ^2^=0.007, *p*=0.854). Although a raised IMD score was associated with an increased likelihood of being identified with fibrosis (OR 1.018, 95% CI 1.00–1.03, *p*=0.018), adjusting for age, gender, BMI, alcohol consumption, and diabetes status, the odds remained similar, which lacked statistical significance (OR 1.014, 95% CI 1.00–1.03, *p*=0.094). However, PCPs in neighborhoods with high deprivation had a mean fibrosis detection rate of 23.6%, while detection rates were significantly lower in practices with medium (11.9%) and low (9.3%) deprivation catchment areas [χ^2^ (2, 3)=16.14; *p*=0.0003] (Figure 5C).

There are 2,324,947 adults in the GM-D, of which 909,364 (39.1%) live in the 30% most deprived wards. Of these, 424,911 (46.7%) patients have at least one risk factor in their clinical records for liver disease. If a 30% fibrosis detection rate were applied, then 127,473 new patients would be identified with a raised LSM requiring further investigation.

## DISCUSSION

Socioeconomic deprivation is a well-established driver of multimorbidity and CLD, including SLD.^2^^,^^18^^–^^20^ Understanding how deprivation influences liver disease burden is essential for reducing health inequalities and improving resource allocation.^22^ In this real-world study based in Greater Manchester—one of the UK’s most deprived urban areas—liver fibrosis detection was highest in patients from the most socioeconomically deprived practices. While risk factors were prevalent across all population groups, nearly one-third of patients in high-deprivation areas had elevated LSMs, compared with <10% in low-deprivation areas (Figure 5).

Our findings align with national and international data demonstrating the disproportionate impact of CLD in communities with higher deprivation. Liver disease mortality is increased 4-fold in the most deprived areas of England.^21^ Over one-third of patients in a cirrhosis surveillance clinic database resided in the most deprived decile and disproportionately experience significant health inequalities^22^^,^^23^ and complications of cirrhosis.^3^^,^^24^ However, this study is the first to specifically evaluate the influence of deprivation on community-based fibrosis detection.

At the individual level, deprivation and fibrosis detection were only significant at the extreme ends of the spectrum, suggesting limited value for individual risk stratification. In contrast, neighborhood-level deprivation (at ward or practice level) was strongly associated with diagnostic yield, suggesting that targeting screening at the population level may be more effective than individual deprivation-based stratification. The difference between individual and locality risk can be explained by the relatively low proportional contribution of traditional risk factors for steatotic liver disease to the social determinants of health contributing to fibrosis (Figure 2). This suggests unidentified factors in the living environment contribute to the risk of liver fibrosis, going beyond health-related risk factors.

Our data suggests proactive screening is no more efficient at identifying high-risk individuals than the traditional referral pathway. However, targeting areas of higher deprivation significantly improves diagnostic yield, despite the lower uptake in these areas. In Greater Manchester, over 100,000 people in the most deprived areas may be at high risk for liver disease. Given the potential scale, long-term strategies need to address the underlying drivers of socioeconomic deprivation and engagement in liver fibrosis detection pathways to reduce the burden of disease.

These findings have significant implications for health service planning. Targeted, scalable, community-level screening can identify liver disease earlier and reduce the risk of progression through timely intervention. However, such pathways do not address the underlying social determinants that drive CLD and multimorbidity. This is more complex than simple community screening initiatives. It requires understanding and addressing the barriers to engagement, alongside coordinated local and national policies to address health inequalities.

There are several possible mechanisms to explain the association between deprivation and fibrosis.^25^ Our case finding pathway dichotomized risk factors. This ignores disease severity, treatment response, adherence, and access to healthcare. These factors may contribute to increased fibrosis prevalence. Our study corroborates that socioeconomic deprivation is associated with higher rates of T2DM and obesity.^26^^,^^27^ The “alcohol harm paradox” describes the increased alcohol-associated morbidity and mortality in lower socioeconomic groups despite similar alcohol consumption across all groups.^28^^,^^29^ The co-occurring metabolic risk factors and health behaviors could help explain this observation.^30^^,^^31^ Poor diet, including high consumption of ultra-processed foods, and reduced access to healthy food options may further augment risk.^32^^–^^34^

This study’s strengths lie in its real-world data involving patients within primary care with a range of etiologies and mixed risk factors. This makes the study more applicable with less bias over highly curated, biopsy-proven cohorts. In addition, the ID-LIVER program was situated in one of the UK’s most deprived and ethnically diverse regions, increasing the relevance of our findings for other urban populations with similar socioeconomic challenges.^2^

IMD offers a multidimensional measure of socioeconomic deprivation, providing a numeric score. It is used widely in the United Kingdom and extrapolates to area-level deprivation rather than reflecting an individual’s socioeconomic status. For the latter, alternative metrics such as household income, associated with HCC diagnosis and curative treatment, would be needed.^35^

Limitations include reliance on primary care coding, which may underreport key risk factors, particularly alcohol use.^36^ Individuals not registered with a PCP, including those experiencing homelessness, who are at elevated risk, may have been unintentionally excluded.^37^ Although inclusion criteria for both pathways were nondependent on risk stratification scores such as FIB-4, there may have been referral bias in the standard-of-care pathway, as primary care physicians may not have referred patients whom they felt to be low risk of fibrosis. Finally, as a study based in a single urban area of the United Kingdom, findings require validation in other settings, both nationally and internationally.

## CONCLUSION

Fibrosis detection is highest in areas of high socioeconomic deprivation, despite risk factors for liver disease being highly prevalent in all areas. As such, targeted screening for liver fibrosis is best performed in areas of high socioeconomic deprivation to maximize detection.

## Supplementary Material
