# Supplementary material for: Targeted community screening in areas of high socioeconomic deprivation enhances the detection of liver fibrosis
Source: Hepatol Commun. 2026 Sep 16;10(10):e1036. doi: 10.1097/HC9.0000000000001036 (PMC13585318; doi:10.1097/HC9.0000000000001036)
Supplement: Supplementary file 1 [file hc9-10-e1036-s001.docx]

Supplementary table 1: Criteria used to identify clinical records with risk factors for steatotic liver disease within the Greater Manchester dataset

| Risk Factor for Steatotic Liver Disease | SNOMED terminology (code) | Directly recorded variable |
| --- | --- | --- |
| Steatosis of the liver | Steatosis of liver (197321007)  Non-Alcoholic Fatty Liver (197315008)  Non-Alcoholic Fatty Liver Disease (1231824009) |  |
| Type 2 Diabetes | Type 2 Diabetes (44054006) |  |
| Harmful Alcohol Intake | Problem drinker (228281002)  Moderate drinker (43783005)  Heavy drinker (86933000)  Very heavy drinker (228279004)  Unhealthy alcohol drinking behaviour (10939881000119105)  Alcohol dependence (66590003)  Alcohol abuse (15167005)  Disorder due to alcohol abuse (288021000119107)  Alcohol withdrawal syndrome (191480000)  Alcohol withdrawal delirium (8635005)  History of alcohol withdrawal syndrome (1141000161108)  Alcohol withdrawal-induced convulsion (308742005)  Alcohol withdrawal hallucinosis (191476005)  Alcoholic liver damage (41309000)  Alcoholic fatty liver (50325005) | Alcohol consumption >35 units/week in females or >50 units/week in males |
| Abnormal serum alanine aminotransferase (ALT) |  | ALT >35 IU/L in females or >50 IU/L in males |
| Obesity | Obesity (414916001) | Body Mass Index (BMI) >30kg/m^2^ |

Supplementary Table 2. Whole cohort recruitment data for the 37 wards with >10 patients recruited. Ward level data for IMD score and subdomain as well as households in poverty, air quality, healthy life expectancy in males and females, Childhood (age 10-11) obesity and green space density obtained through public accessible data (www.mappinggm.org.uk). Wards were split into quartiles based on the prevalence of patients with LSM >8.0kPa in each ward. Where applicable, data is presented as n (% of patients recruited per individual ward). LSM: Liver stiffness measurement; T2DM: Type 2 diabetes mellitus; IMD: Index of Multiple deprivation.

| **Ward** | **Quartile** | **Patients recruited (n)** | **LSM >8.0kPa (% patients recruited** | **T2DM (% patients recruited)** | **Obesity (% patients recruited)** | **Alcohol (% patients recruited)** | **IMD score** | **IMD subdomain** | | | | | | | **Households in poverty** | **Air Quality** | **Healthy Life Expectancy** | | **Age 10-11 obesity** | **Green space** |
| --- | --- | --- | --- | --- | --- | --- | --- | --- | --- | --- | --- | --- | --- | --- | --- | --- | --- | --- | --- | --- |
|  |  |  |  |  |  |  |  | **Income** | **Employment** | **Education** | **Health** | **Crime** | **Housing** | **Environment** |  |  | **Male** | **Female** |  |  |
| **Bowden** | 1 | 28 | 1 (3.57) | 6 (21.43) | 11 (39.29) | 2 (7.14) | 5.65 | 0.04 | 0.03 | 1.06 | -1.14 | -0.39 | 20.3 | 12.29 | 14.02 | 0.21 | 70.82 | 73.02 | 14.12 | -0.9 |
| **Brooklands Trafford** | 1 | 36 | 3 (8.33) | 11 (30.56) | 23 (63.89) | 1 (2.78) | 6.88 | 0.05 | 0.16 | 2.03 | -0.51 | -0.43 | 9.52 | 22.08 | 14.95 | 0.57 | 67.39 | 68.19 | 14.49 | -0.49 |
| **Moss Side** | 1 | 23 | 2 (8.70) | 6 (26.09) | 13 (56.52) | 7 (30.43) | 47.66 | 0.32 | 0.18 | 29.91 | 1.21 | 1.17 | 27.56 | 37.53 | 42.53 | 0.89 | 53.9 | 52.81 | 27.29 | 0.38 |
| **Cheadle and Gatley** | 1 | 57 | 6 (10.53 | 23 (40.35) | 38 (66.67) | 2 (3.51) | 15.23 | 0.08 | 0.08 | 6.63 | 0.12 | 0.69 | 14.75 | 17.39 | 16.49 | 0.6 | 66.14 | 67.33 | 18.48 | -0.2 |
| **Broadheath** | 1 | 75 | 8 (10.67) | 22 (29.33) | 52 (69.33) | 15 (20.00) | 10.06 | 0.07 | 0.06 | 7.95 | -0.27 | -0.41 | 9.95 | 20.86 | 14.95 | 0.44 | 66.8 | 67.06 | 14.61 | -0.1 |
| **Northenden** | 1 | 163 | 20 (12.27) | 40 (24.54) | 99 (60.74) | 15 (9.20) | 42.28 | 0.24 | 0.18 | 32.22 | 1.37 | 1.04 | 24.77 | 24.85 | 24.86 | 0.79 | 57.73 | 59.26 | 23.36 | -0.54 |
| **Withington** | 1 | 24 | 3 (12.50) | 8 (33.33) | 8 (33.33) | 3 (12.50) | 25.11 | 0.11 | 0.07 | 13.51 | 0.94 | 1.22 | 23.96 | 32.08 | 27.18 | 0.68 | 59.56 | 60.23 | 21.46 | 0.03 |
| **Ashton upon Mersey** | 1 | 46 | 6 (13.04) | 18 (39.13) | 28 (60.87) | 7 (15.22) | 9.4 | 0.06 | 0.06 | 2.9 | -0.29 | -0.1 | 8.46 | 23.33 | 13.7 | 0.53 | 65.21 | 67.04 | 12.21 | -0.47 |
| **Hale Central** | 1 | 15 | 2 (13.33) | 3 (20.00) | 8 (53.33) | 3 (20.00) | 6.81 | 0.04 | 0.04 | 0.51 | -0.8 | -0.09 | 14.71 | 22.69 | 11.87 | 0.12 | 72.5 | 72.5 | 7.91 | -0.39 |
| **Levenshulme** | 2 | 15 | 2 (13.33) | 7 (46.67) | 9 (60.00) | 1 (6.67) | 38.66 | 0.21 | 0.13 | 24.19 | 1.03 | 1.24 | 25.04 | 47.74 | 31.41 | 0.89 | 56.32 | 57.28 | 25.14 | 0.65 |
| **Hale Barns** | 2 | 22 | 3 (13.64) | 11 (50.00) | 9 (40.91) | 1 (4.55) | 5.4 | 0.04 | 0.04 | 1.34 | -0.88 | -0.22 | 16.71 | 5.62 | 14.8 | 0.31 | 71.5 | 73 | 15 | -0.9 |
| **Didsbury East** | 2 | 58 | 8 (13.79) | 19 (32.76) | 35 (60.34) | 11 (18.97) | 17.47 | 0.08 | 0.06 | 2.27 | 0.34 | 0.86 | 22.24 | 32.69 | 15.92 | 0.74 | 65.76 | 66.96 | 15.49 | -0.1 |
| **Altrincham** | 2 | 37 | 6 (16.22) | 10 (27.03) | 20 (54.05) | 4 (10.81) | 9.27 | 0.07 | 0.06 | 4.16 | -0.45 | -0.09 | 12.19 | 17.54 | 14.97 | 0.22 | 67.2 | 69.33 | 12.57 | -0.41 |
| **Priory** | 2 | 51 | 9 (17.65) | 26 (50.98) | 32 (62.75) | 8 (15.69) | 11.24 | 0.07 | 0.05 | 2.2 | -0.4 | 0 | 15.83 | 32.34 | 15.13 | 0.67 | 66.43 | 68.13 | 12.97 | -0.74 |
| **Fallowfield** | 2 | 11 | 2 (18.18) | 5 (45.45) | 6 (54.55) | 4 (36.36) | 35.53 | 0.19 | 0.12 | 24.69 | 1.2 | 1.04 | 26.97 | 31.58 | 31.39 | 0.68 | 55.4 | 56.66 |  | -0.44 |
| **Bagueley** | 2 | 114 | 22 (19.30) | 42 (36.84) | 89 (78.07) | 19 (16.67) | 45.94 | 0.26 | 0.2 | 39.74 | 1.74 | 0.83 | 23.11 | 19.02 | 25.6 | 0.8 | 54.96 | 57.12 | 26.7 | 0.1 |
| **Didsbury West** | 2 | 36 | 7 (19.40) | 13 (36.11) | 19 (52.78) | 6 (16.67) | 15.8 | 0.06 | 0.05 | 3.13 | 0.48 | 0.75 | 21.15 | 28.79 | 21.95 | 0.61 | 60.97 | 61.86 | 16.27 | -0.53 |
| **Bucklow** | 2 | 15 | 3 (20.00) | 4 (26.67) | 9 (60.00) | 5 (33.33) | 41.74 | 0.26 | 0.2 | 41.84 | 1.12 | 0.64 | 19.94 | 16.57 | 22.48 | 0.49 | 57.37 | 57.5 | 23.13 | -0.57 |
| **Davyhulme east** | 2 | 15 | 3 (20.00) | 3 (20.00) | 11 (73.33) | 3 (20.00) | 11.94 | 0.07 | 0.07 | 10.8 | -0.08 | -0.39 | 8.55 | 23.2 | 11.1 | 0.78 | 65.33 | 66.63 |  | 0.13 |
| **Gorse Hill** | 3 | 15 | 3 (20.00) | 3 (20.00) | 8 (53.33) | 2 (15.33) | 24.25 | 0.16 | 0.13 | 21.32 | 0.45 | -0.02 | 14.46 | 28.79 | 16.5 | 0.88 | 60.64 | 62.13 | 21.53 | 0.18 |
| **Heald Green** | 3 | 49 | 10 (20.41) | 9 (18.37) | 25 (51.02) | 7 (14.29) | 13.47 | 0.09 | 0.07 | 11.48 | 0.05 | 0.27 | 14.17 | 10.82 | 12.7 | 0.19 | 65.9 | 67.8 | 18.3 | 0.56 |
| **Timperley** | 3 | 23 | 5 (21.74) | 10 (43.48) | 13 (56.52) | 3 (13.04) | 4.89 | 0.04 | 0.03 | 1.93 | -0.67 | -0.49 | 7.73 | 20.37 | 11.03 | 0.41 | 68.93 | 70.37 | 12.98 | -0.51 |
| **Old Moat** | 3 | 27 | 6 (22.22) | 5 (18.52) | 17 (62.96) | 5 (18.52) | 34.03 | 0.18 | 0.11 | 18.98 | 1.23 | 1.35 | 27.21 | 28.71 | 28.24 | 0.62 | 58.19 | 58.32 | 21.83 | -0.26 |
| **Hulme** | 3 | 22 | 5 (22.73) | 9 (40.91) | 16 (72.73) | 3 (13.64) | 32.45 | 0.16 | 0.1 | 18.58 | 1.29 | 1.01 | 25.62 | 33.7 | 45.24 | 0.96 | 52.89 | 56.23 | 29.69 | 0.56 |
| **Whalley Range** | 3 | 13 | 3 (23.08) | 5 (38.46) | 6 (46.15) | 4 (30.77) | 32.91 | 0.22 | 0.14 | 11.66 | 0.92 | 0.69 | 25.61 | 33.84 | 34.95 | 0.73 | 55.99 | 56.11 | 21.66 | -0.59 |
| **Sharston** | 3 | 72 | 17 (23.61) | 28 (38.89) | 45 (62.50) | 11 (15.28) | 49.32 | 0.28 | 0.21 | 44.96 | 1.69 | 1.04 | 24.94 | 20.91 | 28.39 | 0.86 | 54.83 | 54.94 | 27.11 | 0.15 |
| **Ardwick** | 3 | 21 | 5 (23.81) | 9 (42.86) | 13 (61.90) | 2 (9.52) | 47.85 | 0.27 | 0.18 | 35.47 | 1.63 | 0.98 | 29.57 | 28.72 | 45.04 | 0.96 | 51.63 | 52.91 | 27.58 | 0.86 |
| **St Marys** | 3 | 46 | 11 (23.91) | 15 (32.61) | 24 (52.17) | 8 (17.39) | 20.25 | 0.13 | 0.11 | 14.14 | 0.1 | 0.16 | 17.76 | 8.01 | 16.61 | 0.26 | 65.01 | 65.56 | 16.21 | -0.32 |
| **Brooklands Manchester** | 4 | 46 | 11 (23.91) | 16 (34.78) | 21 (45.65) | 8 (17.39) | 35.68 | 0.2 | 0.16 | 26.52 | 1.3 | 0.83 | 22.68 | 20.4 | 22.09 | 0.57 | 58.73 | 59.88 | 21.89 | -0.01 |
| **Village** | 4 | 47 | 13 (27.66) | 16 (34.04) | 29 (61.70) | 12 (25.53) | 16.77 | 0.11 | 0.1 | 12.8 | -0.05 | 0.03 | 13.91 | 14.74 | 15.84 | 0.35 | 64.23 | 66.4 | 16.9 | 0.15 |
| **Sale Moor** | 4 | 32 | 10 (31.25) | 6 (18.75) | 20 (62.50) | 5 (15.63) | 18.15 | 0.12 | 0.1 | 13.34 | 0.07 | 0.2 | 10.99 | 19 | 15 | 0.65 | 64.23 | 66.03 | 17.3 | -0.15 |
| **Burnage** | 4 | 24 | 8 (33.33) | 5 (20.83) | 10 (41.67) | 4 (16.67) | 39.62 | 0.23 | 0.16 | 26.73 | 1.12 | 1.24 | 24.98 | 28.33 | 25.26 | 0.7 | 56.84 | 57.72 | 27.56 | 0.28 |
| **Stretford** | 4 | 11 | 4 (36.36) | 3 (27.27) | 8 (72.73) | 2 (18.18) | 25.35 | 0.15 | 0.12 | 14.7 | 0.41 | 0.65 | 16.83 | 28.02 | 17.5 | 0.84 | 61.7 | 63.77 | 20.97 | -0.19 |
| **Woodhouse Park** | 4 | 56 | 23 (41.07) | 20 (35.71) | 44 (78.57) | 8 (14.29) | 51.05 | 0.29 | 0.22 | 47.05 | 1.84 | 0.87 | 26 | 20.44 | 27.4 | 1.21 | 53.61 | 54.49 | 30.79 | 0.25 |
| **Chorlton Park** | 4 | 18 | 8 (44.44) | 3 (16.67) | 12 (66.67) | 6 (33.33) | 32.65 | 0.18 | 0.13 | 14.49 | 0.91 | 1.14 | 22.09 | 30.87 | 21.99 | 0.57 | 61.62 | 62.72 | 17.49 | -0.57 |
| **Longsight** | 4 | 20 | 9 (45.00) | 9 (45.00) | 12 (60.00) | 2 (10.00) | 47.06 | 0.29 | 0.18 | 40.01 | 1.19 | 1.24 | 25.04 | 32.66 | 39.98 | 0.93 | 53.5 | 54.09 | 27.79 | 0.58 |
| **Gorton and Abbey Hey** | 4 | 11 | 5 (45.45) | 3 (27.27) | 4 (36.36) | 3 (27.27) | 51.23 | 0.29 | 0.2 | 37.61 | 1.33 | 1.66 | 26.26 | 40.14 | 31.9 | 0.93 | 54.11 | 55.72 | 28.64 | 0.11 |

***Supplementary Figure 1: Proportions of patients with LSM >8.0kPa in those who are employed, retired and unemployed****. Significantly higher proportions of patients had a LSM >8.0kPa were seen in those who were retired (23.6%) or unemployed (29.9%) compared to those employed (14.3%)(X^2^ = 20.37, p <0.0001).*

***Supplementary Figure 2: Proportions of patients with LSM <8.0kPa and >8.0kPa determined by Standard Occupation Classification 2020 (SOC2020).*** *Significantly higher proportions of patients had a LSM >8.0kPa in Skilled trade occupations (Group 5; 20.3%), leisure and service occupations (Group 7; 24.4%) and process, plant and machinery operatives (Group 8; 23.7%) (X^2^ = 4.66, p = 0.031).*

**Supplementary Table 3:** Decomposition of Wagstaff’s Concentration Index for the detection of liver fibrosis (Liver Stiffness Measurement (LSM) >8.0kPa) in the ID-LIVER cohort. Negative values indicate LSM >8.0kPa is more concentrated in individuals living in neighborhoods with high socioeconomic deprivation.

| Variable | Absolute contribution to whole cohort concentration index | Relative contribution to whole cohort concentration index, (%) | Elasticity | Individual factor concentration index, (95% CI) |
| --- | --- | --- | --- | --- |
| Age | 0.0140 | -11.2 | 0.588 | 0.024 (0.017 – 0.031) |
| Male sex | 0.004 | -3.55 | 0.103 | 0.043 (0.015 - 0.071) |
| Obesity | -0.004 | 3.44 | 0.168 | -0.025 (-0.049- -0.002) |
| Harmful alcohol use | -0.004 | 3.10 | 0.190 | -0.020 (-0.091 – 0.050) |
| Type 2 Diabetes | -0.017 | 13.8 | 0.762 | -0.022 (-0.033 - -0.012) |

**Supplementary Table 4:** Recruitment to the Proactive cohort by Primary Care Practice (PCP)

| Practice deprivation classification | PCP | Eligibility by PCP (n) | Proportion recruited of those eligible, by PCP (%) | Mean proportion recruited of those eligible, by practice deprivation classification (%, SD) |
| --- | --- | --- | --- | --- |
| Low | St Johns Medical Centre | 1541 | 4.6% | 6.1% (SD ± 1.46) |
|  | West Timperley Medical Centre | 865 | 7.5% |  |
|  | Washway Road Medical Centre | 1898 | 6.3% |  |
| Medium | Didsbury Medical Centre | 1000 | 8.6% | 9.3% (SD ± 0.45) |
|  | Northenden Group Practice | 2181 | 9.5% |  |
| High | The Maples Medical Centre | 1356 | 6.7% | 5.0% (SD ± 2.66) |
|  | The Arch Medical Centre | 1153 | 2.9% |  |
